# Assessing *N. gonorrhoeae* prevalence and testing capacity for new treatment rollout: a scoping review for Cambodia, Thailand, Vietnam and South Africa

**DOI:** 10.1186/s12879-026-13351-1

**Published:** 2026-04-22

**Authors:** Matilda Subek Simon, Alla Babekr, Moaaz A. Elhadad, João Pires, Mathias Nsiah, Fernando Pascual Martinez, Teri Roberts, Sabine Dittrich

**Affiliations:** 1 Deggendorf Institute of Technology, Global Public Health, European Campus Rottal Inn, Pfarrkirchen, Germany; 2 Department for Infection Control Registries, Norwegian Institute of Public Health, Oslo, Norway; 3 Global Antibiotic Research and Development Partnership (GARDP), Geneva, Switzerland; 4 Centre for Tropical Medicine and Global Health, University of Oxford, Oxford, UK

**Keywords:** STI, Sexually transmitted diseases, *Neisseria gonorrhoeae*, *N. gonorrhoeae*, Gonorrhea, Gonorrhoea, Implementation support, Stakeholders, Support

## Abstract

**Background:**

*Neisseria gonorrhoeae*, a major cause of sexually transmitted bacterial infections, poses a significant public health challenge, particularly due to rising antimicrobial resistance (AMR). Regions such as South-East Asia and Africa may face substantial and possibly rising burdens of gonorrhoea, though the full extent remains unclear due to limited diagnostic testing, underreporting, and gaps in aetiological and antimicrobial resistance surveillance – especially in countries lacking population-based prevalence estimates. Addressing deficiencies in data from these regions, particularly those with limited diagnostic infrastructure that are underrepresented in population-based studies, is critical for advancing global sexually transmitted infections (STIs) management and the implementation of new treatments.

**Methods:**

This scoping review focused on the prevalence and reported resistance patterns of *N. gonorrhoeae* across four target countries: Cambodia, Thailand, Vietnam and South Africa. A systematic literature search using PubMed and WHO reports from 2019 to 2023 was conducted, with studies screened based on predefined inclusion and exclusion criteria. Data on prevalence, susceptibility profiles, and laboratory methodologies were extracted and analysed.

**Results:**

Of the 217 articles identified, 29 were included, with South Africa contributing 83% of the studies. No eligible studies were identified from Cambodia. Prevalence rates varied, with the highest observed in one study among men with urethral discharge syndrome in South Africa (87.6% CI95% 85.2–89.9%). Antibiotic susceptibility data were limited to a small subset of articles (4/29, 13.8%), with resistance ranging from ≈78% for ciprofloxacin to ≈1% for cefixime. Laboratory methods primarily employed nucleic acid amplification testing, with limited use of phenotypic testing anywhere, hindering comprehensive AMR monitoring.

**Conclusion:**

This scoping review confirms substantial data gaps on *N. gonorrhoeae* prevalence and antimicrobial resistance across the studied regions, with Cambodia contributing no eligible studies and Thailand and Vietnam remaining underrepresented in published literature. National programs and international partners should prioritise sentinel culture capacity and standardised antimicrobial susceptibility testing and genotypic testing to inform treatment guidelines, detect emerging resistance patterns, and inform public health interventions in these high-burden regions.

**Clinical trial number:**

Not applicable.

**Supplementary information:**

The online version contains supplementary material available at 10.1186/s12879-026-13351-1.

## Introduction

Gonorrhea, a sexually transmitted infection (STI) caused by the bacterium *Neisseria gonorrhoeae*, remains a significant public health concern worldwide [1]. This disease is one of the most common bacterial STIs, and the World Health Organisation (WHO) estimated approximately 82.4 million (47.7-130.4 million) new infections globally in 2020 [1–3]. Additionally, a global rise in resistance has been observed for *N*. *gonorrhoeae* and several extensively drug-resistant strains have emerged, further hindering gonorrhea treatment [2, 4]. Understanding the AMR epidemiology of *N*. *gonorrhoeae* in data-scarce areas is fundamental to controlling this pathogen and improving public health outcomes.

Asian and African regions comprise areas where comprehensive data can be challenging to obtain due to underreporting and varying surveillance systems [5], although both regions have reported concerning AMR trends for gonorrhoea [6]. Albeit high overall [2], the incidence of gonorrhoea in the Asia Pacific and African region varies widely between countries due to differences in healthcare infrastructure, socioeconomic factors, and cultural norms around sexual behaviour [7]. Countries with rapid urbanisation and inadequate sexual health education often experience higher gonorrhoea rates [8].

In 2016, the World Health Organization (WHO) recommended dual therapy with 250 mg ceftriaxone and 1 g azithromycin in settings where AMR information is not available, and monotherapy with 250 mg ceftriaxone in settings where local AMR data confirm susceptibility, for the treatment of uncomplicated gonorrhoea, whereas the 2024 update recommended an increased dose of 1 g ceftriaxone as a single treatment for genital, anorectal and oropharyngeal gonococcal infections [9–11]. Locally adapted treatment guidelines for gonorrhoea can vary regionally, depending on clinical efficacy and resistance trends, but they may include either a single dose of ceftriaxone, cefixime, or spectinomycin or a dual regimen comprising ceftriaxone plus azithromycin or cefixime plus azithromycin [10]. However, the emergence of AMR against these antibiotics poses a significant challenge to the effective treatment of gonorrhoea in Asia and Africa. Furthermore, alarming AMR rates have primarily been attributed to the misuse of antibiotics and easy over-the-counter access in these regions [2]. Current antibiotics indicated for the treatment of *N. gonorrhoeae* are also used for a range of other indications, which may also increase the risk for antibiotic misuse or undertreatment of undiagnosed gonorrhoea. The diminishing treatment options, coupled with the potential of misdiagnosis due to the use of a syndromic management approach, have led to higher rates of untreated or inappropriately treated gonorrhoea, potentially resulting in severe complications, and individual as well as public health consequences for reproductive, maternal, and newborn health [12], that can include infertility, sustained inflammation and chronic pain in women, first trimester abortions, severe neonatal eye infections, and an increased risk of HIV acquisition [10, 13]. For these reasons, the WHO lists *N. gonorrhoeae* as one of its priority pathogens for expedited drug product development [13], and two new antibiotics may be available soon [14]. To inform trial design, demand projections, post-market surveillance strategies and potential guideline updates, it is important to understand the existing knowledge base in terms of resistance, diagnostic use and treatment and testing guidelines.

This review aims to understand the existing evidence in peer-reviewed literature regarding the prevalence and antibiotic resistance profiles for *N. gonorrhoeae* in the four selected countries (Cambodia, Thailand, Vietnam and South Africa) to support implementation and prioritisation efforts for the newly emerging antibiotics [15].

## Methodology

### Country selection

For this scoping review, a subset of the overall possible target countries was selected to enable optimisation of the methodology and data analysis on a smaller set of data. Priority was given to countries included in the Enhanced Gonococcal Antimicrobial Surveillance Program (EGASP) [16] and potentially relevant for the roll-out of new treatments. Countries meeting these criteria were reviewed and narrowed down further by the GARDP team (TR, FPM) with a secondary focus on countries included in the Phase 3 trial for zoliflodacin (Thailand and South Africa). Two other countries in Asia where AMR has been detected were also included, Cambodia and Vietnam, resulting in a final list as follows: Cambodia, Thailand, Vietnam and South Africa.

### Data sources and search criteria

Searches were conducted on PubMed to identify relevant studies published in peer-reviewed journals. Furthermore, publicly available reports published by the WHO were consulted for relevant data. Keywords were kept deliberately nonspecific to ensure all relevant data were identified and captured in the searches. The following keywords were used to obtain prevalence or other relevant epidemiological data on the infections: (((Country) AND (*Neisseria gonorrhoeae*)) AND ((epidemiology) OR (prevalence) OR (incidence)) AND ((“2019“[Date - Publication]: “2023“[Date - Publication]))) AND (English[Language]). Additionally, the following keywords were used to identify additional publications dealing with AMR more specifically: (((((country) AND (*Neisseria gonorrhoeae*)) AND (English[Language])) AND ((“2019“[Date - Publication]: “2023“[Date - Publication]))) AND (antimicrobial resistance)).

### Inclusion and exclusion criteria

#### Inclusion criteria

- Cross-sectional, cohort, clinical trials, systematic reviews, and meta-analysis including information about the prevalence of *Neisseria gonorrhoeae* (NG) with/without *Chlamydia trachomatis* (CT); using symptoms-based and/or aetiological identification.
- For clinical trials, only baseline data were extracted.
- Only the original study relevant to the scope was included for systematic reviews that included various regions.
- Studies with only *Neisseria* spp. data.
- Literature in the English language to allow all team members to cross-check the data.

#### Exclusion criteria

- Case series and case report studies.
- Data collection before 2013.
- NG and CT not included in the screening.
- Data not disaggregated by pathogens but only reported as one.
- Data not disaggregated by country but only reported as regions.
- No relevant data from the target country (Cambodia, Thailand, Vietnam, South Africa).
- Reporting only data regarding the AMR of NG without the prevalence.

### Quality assessment

Based on previously used quality assurance criteria for systematic reviews [17–20] related to AMR, 12 suitable questions were selected to be applied to all studies to assess the studies’ quality (Table 1). Assessments were conducted by two independent reviewers (AB/MN), and quality assessments were recorded as “yes”, “no” or “unclear”. In case of diverging opinions, a consensus was reached after discussion by the study team (AB/MN/ME/SD).

**Table 1:** Overview of selected quality assessment questions and the source of the question

Quality question	Source
Q1. Is the research design described?	[19]
Q2. Was the sampling frame appropriate to address the target population?	[21]
Q3. Does the study state the period of time during which samples were specifically collected?	[19]
Q4. Is the setting of the study and data acquisition clearly described (e.g.: hospital vs. community acquired)?	[19]
Q5. Is the study population clearly described (e.g.: percent children and percent adults, comorbidities)?	[19]
Q6. Was the sample size adequate? *	[21]
Q7. Are the criteria for enrolment in the study clearly stated?	[19]
Q8. Were valid methods used for the identification of the condition? **	[21]
Q9 Does the study describe the number of samples tested and indicate reasons for exclusion, if any?	[19]
Q10. What is the number of samples that were tested (excluding discarded samples)?	[19]
Q11. Does the study describe isolates by category including sex and age?	[19]
Q12. Was there appropriate statistical analysis?	[21]

### Data collection and analysis

#### Standardised data collection

Initial title and abstract screenings were performed by individuals (ME/NM/AB) using the collaboration tool RAYYAN (https://www.rayyan.ai/) [22]. Any queries were brought to weekly team meetings (SD/ME/NM/AB) and resolved by discussion and majority agreement. Data were extracted (ME/NM/AB) using a standardised form on Google Forms. Sample type and infection site were gathered along with prevalence data, standardised population information, antibiotic susceptibility testing (AST) and information about the site of infection. After completion, the data were downloaded into an Excel form, cleaned, and further processed using Excel functionalities.

#### Data analysis and reporting

Analysis and visualisation were performed using Excel (Microsoft) and R Statistical Software.

To obtain the 95%CI for all prevalence estimates when not provided by the original manuscript, an external online tool was used (reported without continuity correction [23]), where values were missing in the publications (http://vassarstats.net/prop1.html).

## Results

### General data retrieval – PRISMA

**Fig. 1:**
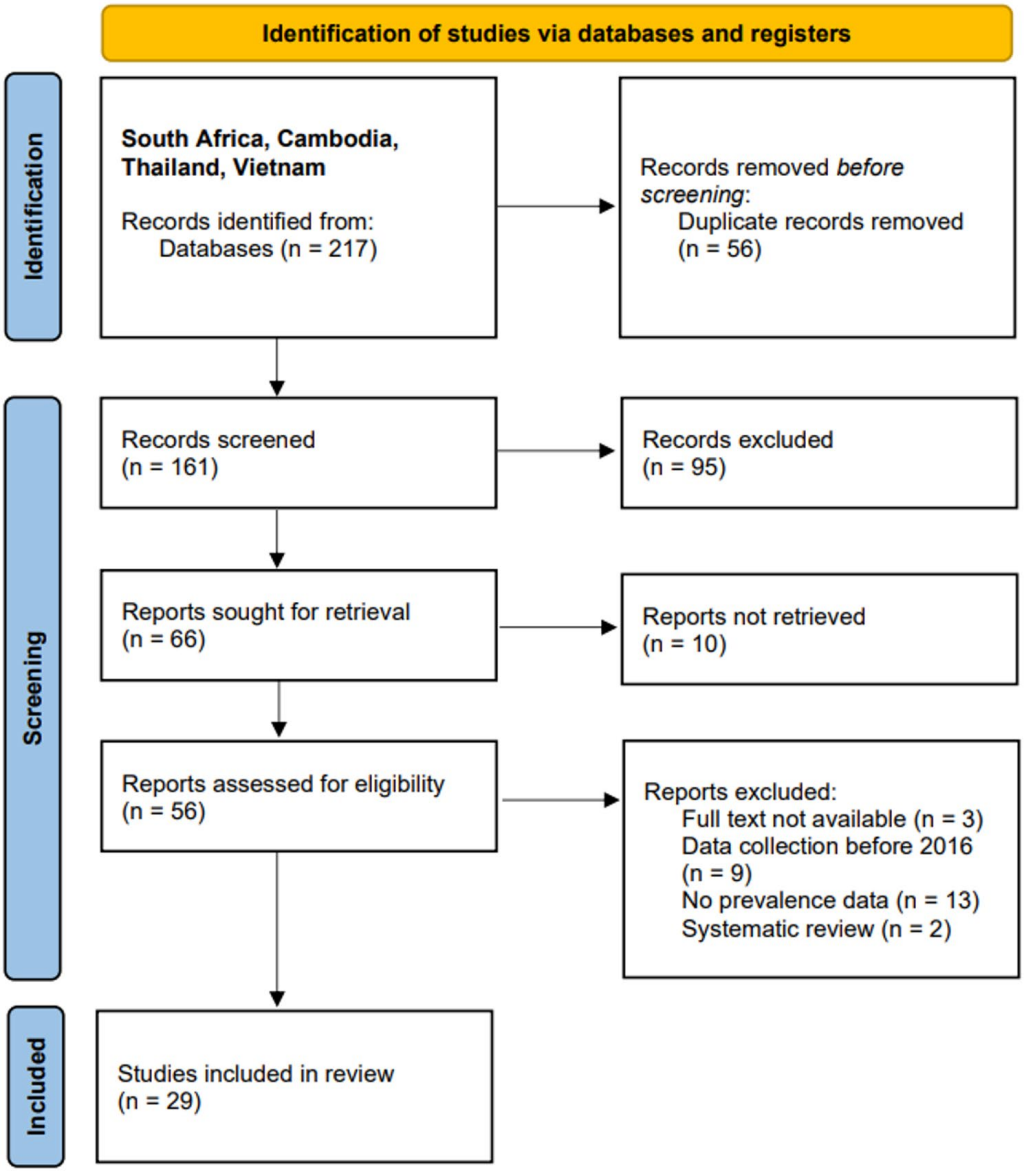
PRISMA diagram representing all data identified and included/excluded for the target countries

In total, 217 articles were identified in the search, of which only 29 (13.3%) were included in the final data extraction. A PRISMA diagram was developed, which represents all inclusions/exclusions and where they occurred [24] (Fig. 1).

### Overview of studies

#### Available data by year, country, and population

Overall, most data were available from South Africa, with 24 studies (24/29, 83%), and no study that met the inclusion criteria was available from Cambodia (0/29, 0%). One (1/29, 3%) and four (4/29, 14%) studies were identified from Thailand and Vietnam, respectively. Many studies (9/29, 31%) were published in 2022 (from South Africa and Vietnam). Studies were performed in adolescent populations (men/women) (*n* = 3), MSM (*n* = 5), pregnant women (both - living and not living with HIV) (*n* = 7), other, sexually active women (*n* = 3); sexually active (*n* = 2), non-pregnant women attending sexual and reproductive services (*n* = 1)), women attending health care facilities for cervical cancer screening (*n* = 2) or people belonging to other relevant groups (*n* = 7; attending for UDS (*n* = 3), HIV-negative (*n* = 3), transgender woman (*n* = 1)). In one case, the population was not mentioned. In only one study from South Africa was the specific population not identified. Despite the wide range of populations studied (Table 2), only South Africa had data across all population groups, while Thailand and Vietnam only had data for MSM and/or transgender populations. In none of the Asian countries was data from the general populations identified in the search (Supplementary Table 1).

#### Age and gender distribution

Studies covered participants aged 15–65 years, with most studies focusing on participants above 18 years of age (both men and women) (12/29, 41%). All studies investigating only women (15/29, 52%) included only women of reproductive age. Three studies that investigated both sexes did not specify the age group they included (Supplementary Table 2). Females were studied most frequently (52%); a further 17% focused on male participants (5/29) only, 17% on both (5/29), 14% investigated transgender women (1/29) and both transgender men and women (1/29).

#### Data quality

All studies were assessed for quality using the predefined and validated quality questions (Supplementary Table 3). Overall, the quality of studies, based on these criteria, appeared to be high, indicating that the study designs were appropriate for the question at hand. For most studies, all questions were answered with “yes” (19/29, 66%), and only a minority had more than one of the questions answered with “no” or “unclear” (4/29, 14%). The latter category mainly refers to the appropriateness of the statistical analysis and the sample size.

### Prevalence of gonorrhoea infections

Overall, the reviewed studies investigated a total of 18,574 patients across all populations, with most patients investigated in South Africa (10,297/18,574; 55.4%). A total of 43.8% (8,135/18,574) and 0.8% (142/18,574) of patients were included from Vietnam and Thailand, respectively. In South Africa, the studies with the most participants belonged to populations with urethral discharge syndrome (1,768/10,297; 17.9%), people not living with HIV- (1,685/10,297; 16.1%), and pregnant women (1,530/10,297; 14.9%). In Vietnam, 78% (7786/8,135) of participants were MSM, and in Thailand, transgender women were the only population studied. (Table 2).

**Fig. 2:**
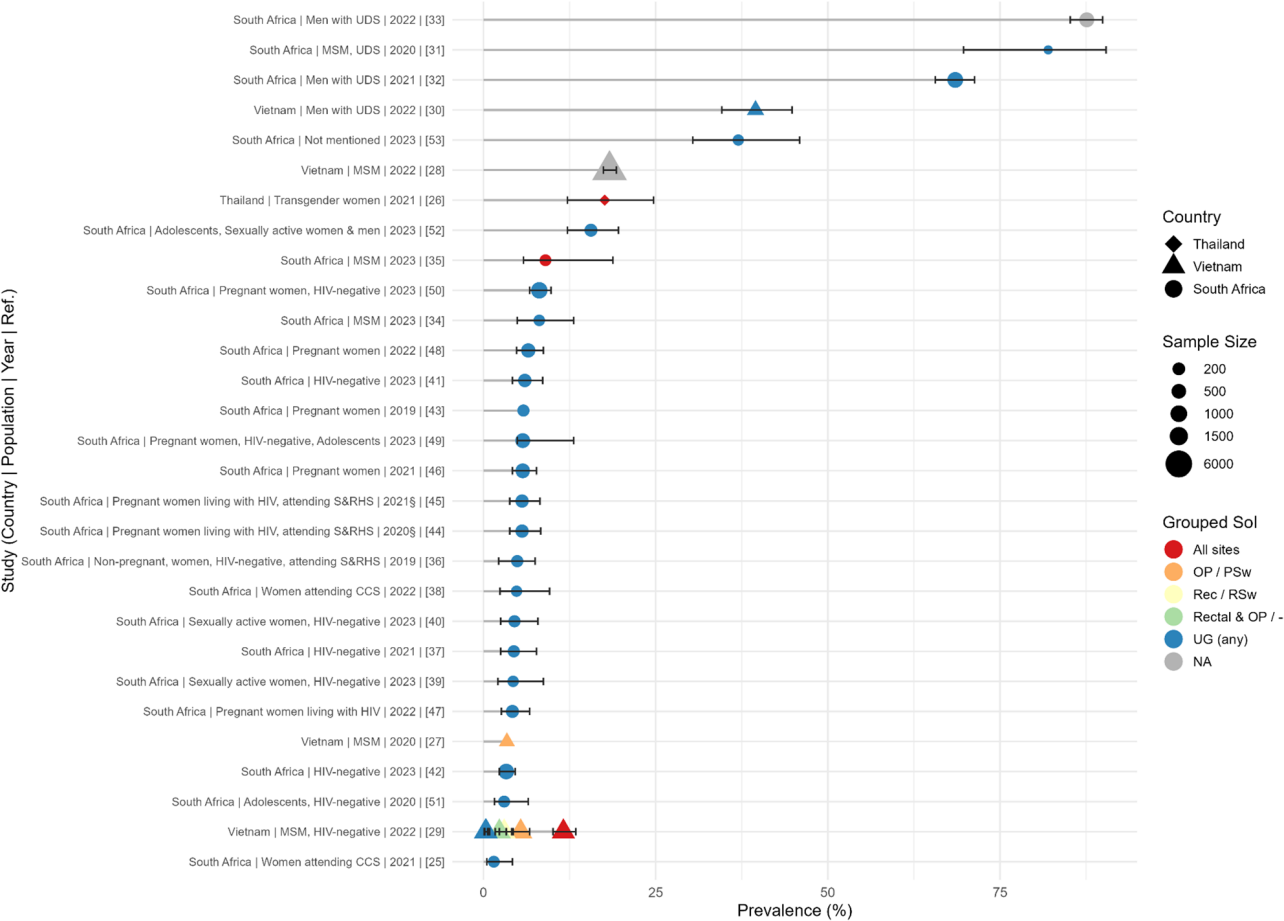
Overview of *Neisseria gonorrhoeae* prevalence in the different studies, countries and sample sites of infection. Data are presented from highest to lowest prevalence (black bars represent the 95%CI, as reported in Table 2) with countries represented by three different shapes (diamond: Thailand, triangle: Vietnam, circle: South Africa). Further, the size of the study is proportional to the size of the icon, while the colour indicates the site of infection (red: all sites, orange: oropharyngeal (OP), yellow: rectal, green: OP and rectal, blue: urogenital, all sample types, grey: data on site of infection not available). Detailed sample size and additional details about the population can be found in Table 2. PSw: Pharyngeal Swab, RSw: Rectal Swab; SoI: Site of Infection

Across all studies, the prevalence ranged from around 1.5% to 87.6% depending on the population and country (Fig. 2). The highest NG prevalence was reported in MSM with UDS from Cape Town, South Africa (87.6%). The NG prevalence in pregnant women reported in studies (*n* = 8) from South Africa (in people both living and not living with HIV) ranged from 4.2% to 8.1%. The lowest measured prevalence (1.5% CI95%: 0.5–4.2) was reported in one study [25] in South Africa investigating patients attending cervical cancer screening.

#### Antimicrobial susceptibility testing for *Neisseria gonorrhoeae*

AST data were only available in a small subset of the articles (4/29, 13.8%; Table 3), with the proportion of resistant samples varying from 100% (ciprofloxacin) to approximately 1% (cefixime).

**Table 2:** Overview of prevalence estimates (NG Prev) in the included studies, including site of infection (SoI) and sample type used

Year	Population Group	Sample size	NG Prev	CI (95%)	SoI / Sample	Ref
**Thailand**						
**Transgender**						
2021	Transgender women	142	17.6%	12.2–24.7**	All	[26]
		-	-	-	UG / U	
		-	-	-	Rec / RSw	
		-	-	-	OP / PSw	
**Vietnam**						
2020	MSM	207	3.4%	NA	OP / PSw	[27]
2022	MSM	6090	18.3%**	17.4–19.3**	-	[28]
2022	MSM, HIV-negative	1489	11.60%	10.1–13.4**	All sites	[29]
		1486	5.4%	4.3–6.7**	OP / PSw	
		1419	3.0%	2.3–4.1**	Rec / RSw	
		1480	0.3%	0.1–0.6**	UG / U	
		1412	0.3%	0.1–0.7**	UG & Rectal / -	
		1477	0.3%	0.1–0.7**	UG & OP / -	
		1416	2.3%	1.7–3.3**	Rectal & OP / -	
		1409	0.4%	0.2–0.9	Rec&OP&UG / -	
2022	Men with UDS	349	39.50%	34.6–44.8**	UG / UrSw	[30]
**South Africa**						
***MSM / UDS Men***						
2020	MSM, UDS	51	82%	69.7–90.4**	UG / U, UrSw	[31]
2021	Men with UDS	1000	68.5%**	65.6–71.3**	UG / UDSw	[32]
2022	Men with UDS	768	87.6%	85.2–89.9	-	[33]
2023	MSM	173	8.1%	4.9–13.1	UG / U	[34]
2023	MSM	200	9%	5.8–18.8**	All	[35]
		-	-	-	UG / GeSw	
		-	-	-	Rec / RSw	
		-	-	-	OP / OrSw	
***Women (General / HIV-negative)***						
2019	Non-pregnant, women, HIV-negative, attending S&RHS	247	4.90%	2.2–7.5	UG / VSw	[36]
2021	HIV-negative	248	4.40%	2.5–7.7**	UG / VSw	[37]
2021	Women attending CCS	205	1.50%	0.5–4.2**	UG / CSw	[25]
2022	Women attending CCS	145	4.80%	2.4–9.6**	UG / CSw	[38]
2023	Sexually active women, HIV-negative	162	4.3%	2.1–8.7**	UG / VSw	[39]
2023	Sexually active women, HIV-negative	243	4.50%	2.5–7.9**	UG / VSw	[40]
2023	HIV-negative	451	6.0%	4.2–8.6**	UG / VSw, CSw	[41]
2023	HIV-negative	959	3.3%	2.3–4.6**	UG / U, CSw, VSw	[42]
***Pregnant Women***						
2019	Pregnant women	242	5.80%	NA	UG / VSw	[43]
2020^§^	Pregnant women living with HIV, attending S&RHS	427	5.60%	3.8–8.3	UG / VSw	[44]
2021^§^	Pregnant women living with HIV, attending S&RHS	427	5.6%	3.8–8.2**	UG / VSw	[45]
2021	Pregnant women	669	5.7%	4.2–7.7**	UG / VuVaSw	[46]
2022	Pregnant women living with HIV	385	4.2%	2.6–6.7**	UG / VSw	[47]
2022	Pregnant women	619	6.5%	4.8–8.7**	UG / VuVaSw	[48]
2023	Pregnant women, HIV-negative, Adolescents	752	5.7%	4.9–13.1	UG / VSw	[49]
2023	Pregnant women, HIV-negative	1194	8.1%	6.7–9.8**	UG / VSw	[50]
***General / Mixed / Not Specified***						
2020	Adolescents, HIV-negative	216	3%	1.6–6.5**	UG / U, VSw	[51]
2023	Adolescents, Sexually active women & men	366	15.6%	12.2–19.6**	UG / U, VSw	[52]
2023	Not mentioned	148	37.%	30.4–45.9**	UG / UgSw	[53]

Studies are grouped by country, population group and ordered by publication year and sample size. “-“: data not available; *Year of publication

** Not all studies included 95%CI and those were separately calculated (95%CI calculated separately (http://vassarstats.net/prop1.html) [23];

§ Studies report data from the same population; #Sample size for CT: 685

UDS: Urethral discharge syndrome; MSM: men who have sex with men; CCS: cervical cancer screening; S&RHS: sexual & reproductive health services; Site of Infection related abbreviations: UG: Urogenital site: OP: Oropharyngeal site, Rectal: Rectal site; Sample type related abbreviations: CSw: Cervical Swab, GeSw: Genital Swab, OrSw: Oral Swab, PSw: Pharyngeal Swab, RSw: Rectal Swab, U: Urine, UDSw: Urethral discharge Swab, UgSw: Urogenital Swab, UrSw: Urethral Swab, VSw: Vaginal Swab, VuVaSw: Vulvovaginal Swab; “-“: data not available

**Table 3:** Overview of publications with antimicrobial resistance data included

1st Author (Publication Year)	Country	Study population (Sample Size)	Sample type	Antimicrobial test	Prevalence (Resistant/tested, %)
Dong (2020)	Vietnam	MSM (207)	Pharyngeal Swab	Ciprofloxacin	9/9 (100)
				Cefixime	0/9 (0)
				Ceftriaxone	0/9 (0)
				Cefpodoxime	8/9 (88.9)
Maduna (2020)	South Africa	MSM, UDS (51)	Urethral Swab	Ciprofloxacin	21/27 (77.8)
				Azithromycin	4/27 (15)
				Penicillin	9/27 (33.3)
				Cefixime	0/27 (0)
				Ceftriaxone	0/27 (0)
				Spectinomycin	0/27 (0)
Kularatne (2021)	South Africa	Men, UDS (768)	Urine, Urethral Swab	Cefixime	1/685 (0.2)
				Ceftriaxone	0/685 (0)
Kularatne (2022)	South Africa	Men, UDS (768)	Urine, Urethral Swab	Cefixime	1/542 (0.2)
				Ceftriaxone	0/542 (0)
				Azithromycin	0/542 (0)

### Site of infection

The predominant site of infection from which prevalence data were reported was the urogenital site (Fig. 2; Table 2). Only 3 (3/29, 10.3%) of the studies reported to have taken samples from other sites, namely rectal or pharyngeal swabs. Of the three studies with additional sites sampled, only one provided additional prevalence estimates for infection from rectal (48.0%) and oropharyngeal sites (70.5%).

### Laboratory methodologies

**Fig. 3:**
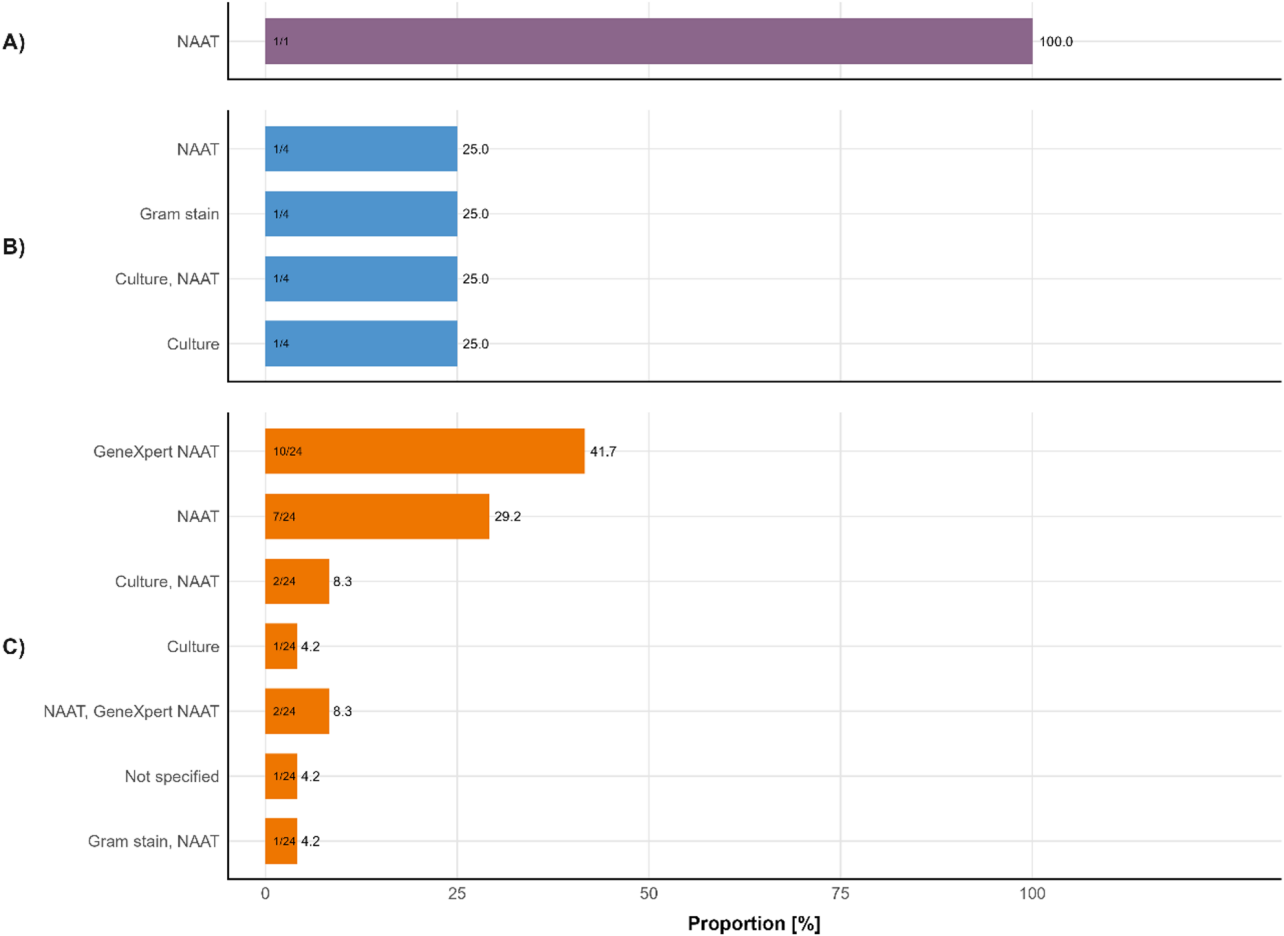
Overview of all laboratory methodologies used to identify *N. gonorrhoeae*. **A**) Thailand. **B**) Vietnam. **C**) South Africa; NAAT: Nucleic acid amplification test, GeneXpert NAAT: CT/NG cartridge run on GeneXpert device

To understand the mode of testing in the different studies, and to approximate the ability to conduct resistance monitoring after identification (either phenotypic or genotypic), the different methods were collected and summarised (Fig. 3). Overall, very few classical microbiological methods, which would allow phenotypic testing (culture or Gram staining), were used (7/29, 24%). The majority (12/29, 41%) of studies report having used nucleic acid amplification tests (NAATs) for identification, either in the form of commercial or research-use-only quantitative PCR assays or the Xpert CT/NG NAAT (9/12, 75%), a near-patient test that is run on the GeneXpert instrument (Cepheid, Danaher). One study (3%) did not report the detection method.

## Discussion

This scoping review assessed 29 studies identified in the published literature on the prevalence of *N. gonorrhoeae* across four target countries: Cambodia, Thailand, Vietnam, and South Africa. South Africa contributed the most data, and no studies meeting the inclusion criteria were available from Cambodia. An increase in publication frequency in the public domain was observed over time, indicating a growing research focus in these regions. Men with urethral discharge syndrome (UDS) demonstrated the highest prevalence rates of *N. gonorrhoeae*. These findings highlight that symptomatic populations, particularly those with urethral discharge, show substantially higher prevalence rates than general populations. Without data from general population proxies, surveillance systems lack essential information to accurately estimate the prevalence of *N. gonorrhoeae* across all sexually active adults. This gap restricts our ability to generalise findings or establish regional profiles of *N. gonorrhoeae* transmission and AMR, which are crucial for implementing effective public health interventions [54].

The WHO has traditionally recommended reviewing treatment guidelines when resistance exceeds 5% [2], highlighting the need for systematic AMR monitoring to inform clinical guidelines. In our review, only 13.8% of studies provided resistance data, creating a critical surveillance gap.

The recent analysis of surveillance data reported to the WHO in 2019–2022, overlapping with our inclusion timeline, also highlights an increase in reported isolates with resistance or decreased susceptibility to cefixime, azithromycin, and ciprofloxacin [55]. This analysis also reports data from our target countries, with Cambodia reporting that ≥5% or more isolates were resistant or showed decreasing susceptibility to ceftriaxone, and Thailand and South Africa reporting fewer than 5% or none of the isolates being resistant or showing reduced susceptibility in 2022 [55]. The same analysis confirms persistently high ciprofloxacin resistance (≥90%) in both Thailand and Cambodia [55], demonstrating that this antibiotic is no longer a viable treatment option in these settings. Country-specific published data align with these trends. A systematic review by Yakobi et al. (2024) showed that South Africa’s Western Cape province had 100% ciprofloxacin resistance in tested isolates, reinforcing the ineffectiveness of this antibiotic in the region [56]. Similarly, Hooshiar et al. (2024) identified a global weighted pooled resistance rate to ciprofloxacin of 51.6% across 68 countries [57]. Our scoping review findings are in line with these global and regional patterns: the single South African study providing resistance data reported 78% ciprofloxacin resistance, aligning with the broader literature indicating near-universal ciprofloxacin resistance across our study countries and beyond.

In addition to ciprofloxacin, concerning patterns of resistance to currently recommended first-line treatments are emerging. WHO EGASP data from 2023 further corroborate these concerning resistance patterns: among nine sentinel countries, ceftriaxone resistance was exclusively found in Cambodia (15.3% of isolates) and Vietnam (20.4% of isolates), with resistance to both ceftriaxone and azithromycin observed in 9% and 2% of isolates, respectively [4]. The consistency between our scoping review findings, published systematic analysis of WHO NG resistance surveillance data, including via the EGASP reports [2, 4, 55, 58], and country-specific reviews [56, 57], validates that published research reflects broader resistance data, where data exist. However, the stark contrast between the limited published data identified in our review (only 4/29 studies reporting AMR) and the substantial EGASP surveillance data focused on resistance, highlights a critical gap between surveillance activities and accessible peer-reviewed literature—particularly for Cambodia and Vietnam, which contributed minimal published data to our review despite having significant documented AMR concerns through EGASP surveillance (which does not publish prevalence data).

Diagnostic approaches across the studied countries rely predominantly on NAAT-based testing. Point-of-care NAAT tools represent a valuable opportunity for increased diagnosis, including the detection of genotypic proxies that correspond to susceptibility profiles. However, the lack of culture-based testing for phenotypic antimicrobial susceptibility monitoring impedes the collection of comprehensive resistance data, particularly for surveillance purposes. This gap may lead to outdated or inaccurate local antibiograms and delayed detection of emerging resistance patterns. Strengthening microbiology laboratory infrastructure, ensuring dedicated funding, and implementing combined testing algorithms with referral pathways (NAAT and culture) would therefore be important for improving the quality and completeness of resistance data.

Our data confirm this predominant focus on molecular methods, the preferred diagnostic test, with only 24% of studies describing the use of classical microbiological methods (culture or Gram staining) for diagnosis, and ultimately only 13.8% of studies providing resistance results. The current study shows that the most frequently sampled clinical site was the urogenital tract, with more than 14,000 relevant samples across different settings, though this may reflect sampling bias rather than true infection distribution. Enhanced sampling from extragenital sites (pharyngeal and rectal) would improve our understanding of *N. gonorrhoeae* distribution across anatomical sites and inform treatment strategies for multi-site infections.

This review has several limitations that impact the study selection and interpretation of its findings. First, the selection of countries was based on convenience and highlights that the primary goal of the data was to help inform treatment roll-out plans. Further, the review relied exclusively on studies available through PubMed and WHO reports (in English; not utilising MeSH terms), potentially excluding relevant data from regional or non-indexed sources. This constraint may limit insights specific to countries with limited peer-reviewed data, such as Cambodia, where localised studies may not be published in the English language. To overcome this for future work, more databases should be included in all local (relevant to the search country) languages and trial databases (like clinicaltrial.gov) should be consulted to avoid publication, indexing and language bias. Further, the lack of extragenital samples in MSM populations might result in surveillance bias, as the number of infections could be underestimated. Additionally, the limited number of countries covered in this review restricts its ability to provide a regionally representative analysis. The heterogeneity in study designs and sampling methodologies across countries further limits direct comparisons and prevents meta-analysis. Certain studies lacked specific site-of-infection data, impeding a precise understanding of *N. gonorrhoeae* distribution across anatomical sites and more standardised reporting and sampling strategies integrated into decentralised testing algorithms could help overcome this in the future.

Despite these limitations, the review provides an overview of gonorrhoea prevalence and AMR in key countries that can help inform public health actions and inform implementation plans for emerging new treatments. Our observations on sampling types, diagnostic test use, and treatment guidelines provide insights into the surveillance landscape across these countries in two major regions (Southeast Asia and Africa) and can help inform trial strategies, post-market surveillance monitoring plans and guideline updates for newer treatments. The substantial variation in the availability of peer-reviewed published data between countries underscores the need for strengthened surveillance systems and strong research capacity to support academic publishing to ensure research evidence availability in peer-reviewed articles.

Further, investment in laboratory capacity for both diagnostic testing and antimicrobial susceptibility testing will be essential for guiding treatment decisions and monitoring the effectiveness of new therapeutic options, both in the early stages of implementation and in the later stages of post-market microbiological surveillance of new treatments.

## Electronic supplementary material

Below is the link to the electronic supplementary material.

Supplementary Material 1

## Data Availability

All data generated or analysed during the study are included in this published article and its supplementary materials.
