# Supplementary material for: Assessing N. gonorrhoeae prevalence and testing capacity for new treatment rollout: a scoping review for Cambodia, Thailand, Vietnam and South Africa
Source: BMC Infect Dis. 2026 Apr 22;26:1086. doi: 10.1186/s12879-026-13351-1 (PMC13235072; doi:10.1186/s12879-026-13351-1)
Supplement: Supplementary file 1 — Supplementary Material 1 [file 12879_2026_13351_MOESM1_ESM.docx]

**Supplementary Table 1: Overview of the different populations studied in different countries.**

| Population label | Cambodia | Thailand | Vietnam | South Africa | Total |
| --- | --- | --- | --- | --- | --- |
| *Adolescent population* | ***0*** | ***0*** | ***0*** | ***3*** | ***3*** |
| Adolescents, HIV-uninfected | 0 | 0 | 0 | 1 | 1 |
| Adolescents, sexually active women / men | 0 | 0 | 0 | 1 | 1 |
| Pregnant women, HIV-uninfected, adolescents | 0 | 0 | 0 | 1 | 1 |
| *Men who have sex with men (MSM)* | ***0*** | ***0*** | ***3*** | ***3*** | ***6*** |
| MSM | 0 | 0 | 2 | 2 | 4 |
| MSM, HIV-uninfected | 0 | 0 | 1 | 0 | 1 |
| MSM, urethral discharge syndrome | 0 | 0 | 0 | 1 | 1 |
| *Pregnant woman / antenatal care (ANC)* | ***0*** | ***0*** | ***0*** | ***7*** | ***7*** |
| Pregnant women | 0 | 0 | 0 | 3 | 3 |
| Pregnant women, HIV-infected | 0 | 0 | 0 | 2 | 2 |
| Pregnant women, HIV-infected, attending sexual and reproductive services | 0 | 0 | 0 | 1 | 1 |
| Pregnant women, HIV-uninfected | 0 | 0 | 0 | 1 | 1 |
| *Sexually active woman* | ***0*** | ***0*** | ***0*** | ***3*** | ***3*** |
| Sexually active women, HIV-uninfected | 0 | 0 | 0 | 2 | 2 |
| Non-pregnant, HIV-uninfected women, attending sexual and reproductive services | 0 | 0 | 0 | 1 | 1 |
| *Symptomatic (men)* | ***0*** | ***0*** | ***1*** | ***2*** | ***3*** |
| Urethral discharge syndrome | 0 | 0 | 1 | 2 | 3 |
| *Other* | ***0*** | ***1*** | ***0*** | ***6*** | ***7*** |
| Attending cervical cancer screening | 0 | 0 | 0 | 2 | 2 |
| HIV-uninfected | 0 | 0 | 0 | 3 | 3 |
| Transgender women | 0 | 1 | 0 | 0 | 1 |
| Population not mentioned | 0 | 0 | 0 | 1 | 1 |
| *Total (all populations)* | ***0*** | ***1*** | ***4*** | ***24*** | ***29*** |

**Supplementary Table 2: Overview of the number of studies in different age groups, broken out by sex.**

| **Age groups studied** | **Women** | **Men** | **Women, Men** | **Total** |
| --- | --- | --- | --- | --- |
| ≥15 | 1 |  |  | 1 |
| ≥16 |  | 2 |  | 2 |
| ≥18 | 7 | 5 |  | 12 |
| ≥30 | 1 | 1 |  | 2 |
| 15-23 |  | 1 |  | 1 |
| 15-65 |  | 1 |  | 1 |
| 16-24 |  |  | 1 | 1 |
| 16-25 | 1 |  |  | 1 |
| 16-35 |  | 1 |  | 1 |
| 18-35 | 1 | 1 |  | 2 |
| 18-40 | 2 |  |  | 2 |
| 19-25, 26-35, 36-45, 46-55 |  | 1 |  | 1 |
| Not mentioned | 2 |  |  | 2 |
| Total | 15 | 13 | 1 | 29 |

**Supplementary Table 3: Overview of all studies (identified by DOI) and the quality questions** (Green=Yes, Orange=Not Clear, Red=No)

| **Year** | **First Author** | **DOI** | **Q.1 Design described?** | **Q.2 Appropriate sampling?** | **Q.3 Sampling period stated?** | **Q.4 Study setting clear?** | **Q.5 Study population clear?** | **Q6 Sample size adequate?** | **Q.7 Enrollment criteria clear?** | **Q.8 Valid identification method?** | **Q.9 Inclusion/exclusion of testing clear?** | **Q.10 Sample size clear ?** | **Q.11 Analysis by categories?** | **Q.12 Appropriate statistical analysis?** |
| --- | --- | --- | --- | --- | --- | --- | --- | --- | --- | --- | --- | --- | --- | --- |
| 2019 | Davey J | 10.1371/journal.pone.0218349 |  |  |  |  |  |  |  |  |  |  |  |  |
| 2019 | Garrett, N | 10.1136/bmjopen-2018-026888 |  |  |  |  |  |  |  |  |  |  |  |  |
| 2020 | Maduna, L. D. | 10.1128/AAC.00906-20 |  |  |  |  |  |  |  |  |  |  |  |  |
| 2020 | Chitneni, P | 10.1177/0956462420915395 |  |  |  |  |  |  |  |  |  |  |  |  |
| 2020 | Medina-Marino, A | 10.1177/0956462419898612 |  |  |  |  |  |  |  |  |  |  |  |  |
| 2020 | Dong, H. V. | 10.1093/cid/ciz365 |  |  |  |  |  |  |  |  |  |  |  |  |
| 2021 | Peters, R. P. H. | 10.1111/1471-0528.16617 |  |  |  |  |  |  |  |  |  |  |  |  |
| 2021 | Nyemba, D. C. | 10.1136/sextrans-2020-054631 |  |  |  |  |  |  |  |  |  |  |  |  |
| 2021 | Taku, O | 10.7717/peerj.10793 |  |  |  |  |  |  |  |  |  |  |  |  |
| 2021 | Kularatne, R. S. | 10.1128/AAC.00389-21 |  |  |  |  |  |  |  |  |  |  |  |  |
| 2021 | Hiransuthikul, A | 10.1002/jia2.25801 |  |  |  |  |  |  |  |  |  |  |  |  |
| 2021 | Mngomezulu, K | 10.3389/fimmu.2021.695201 |  |  |  |  |  |  |  |  |  |  |  |  |
| 2022 | Kularantne, R | 10.1097/OLQ.0000000000001647 |  |  |  |  |  |  |  |  |  |  |  |  |
| 2022 | Nyemba, D.C. | 10.1186/s12884-022-04520-6 |  |  |  |  |  |  |  |  |  |  |  |  |
| 2022 | Trinh, T. M. | 10.3201/eid2802.211788 |  |  |  |  |  |  |  |  |  |  |  |  |
| 2022 | Nguyen, B. H. | 10.1080/13685538.2022.2063272 |  |  |  |  |  |  |  |  |  |  |  |  |
| 2022 | Adamson, P. C | 10.1097/OLQ.0000000000001626 |  |  |  |  |  |  |  |  |  |  |  |  |
| 2022 | Taku, O | 10.1128/spectrum.02229-21 |  |  |  |  |  |  |  |  |  |  |  |  |
| 2022 | Ngobese, B | 10.1155/2022/7930567 |  |  |  |  |  |  |  |  |  |  |  |  |
| 2023 | Malefo, M. A. | 10.4102/PHCFM.V15I1.4080 |  |  |  |  |  |  |  |  |  |  |  |  |
| 2023 | Mashingaidze, R | 10.1371/journal.pgph.0001782 |  |  |  |  |  |  |  |  |  |  |  |  |
| 2023 | Govender, V | 10.1136/sextrans-2022-055658 |  |  |  |  |  |  |  |  |  |  |  |  |
| 2023 | Harryparsad, R | 10.1371/journal.pone.0294285 |  |  |  |  |  |  |  |  |  |  |  |  |
| 2023 | Marcus, R | 10.1186/s12913-023-10068-8 |  |  |  |  |  |  |  |  |  |  |  |  |
| 2023 | Qulu, W | 10.1371/journal.pone.0294698 |  |  |  |  |  |  |  |  |  |  |  |  |
| 2023 | Moodley, C | 10.1371/journal.pone.0292534 |  |  |  |  |  |  |  |  |  |  |  |  |
| 2023 | Delany-Moretlwe, S | 10.1136/sextrans-2022-055696 |  |  |  |  |  |  |  |  |  |  |  |  |
| 2023 | Asare, K | 10.1093/cid/ciac824 |  |  |  |  |  |  |  |  |  |  |  |  |
